# Human spongiosa mesenchymal stem cells fail to generate cardiomyocytes *in vitro*

**DOI:** 10.1186/1477-5751-8-11

**Published:** 2009-11-10

**Authors:** Svetlana Mastitskaya, Bernd Denecke

**Affiliations:** 1 Interdisciplinary Centre for Clinical Research (IZKF) "BIOMAT.", RWTH Aachen University, Aachen, Germany

## Abstract

**Background:**

Human mesenchymal stem cells (hMSCs) are broadly discussed as a promising cell population amongst others for regenerative therapy of ischemic heart disease and its consequences. Although cardiac-specific differentiation of hMSCs was reported in several *in vitro *studies, these results were sometimes controversial and not reproducible.

**Results:**

In our study we have analyzed different published protocols of cardiac differentiation of hMSCs and their modifications, including the use of differentiation cocktails, different biomaterial scaffolds, co-culture techniques, and two- and three-dimensional cultures. We also studied whether 5'-azacytidin and trichostatin A treatments in combination with the techniques mentioned above can increase the cardiomyogenic potential of hMSCs. We found that hMSCs failed to generate functionally active cardiomyocytes *in vitro*, although part of the cells demonstrated increased levels of cardiac-specific gene expression when treated with differentiation factors, chemical substances, or co-cultured with native cardiomyocytes.

**Conclusion:**

The failure of hMSCs to form cardiomyocytes makes doubtful the possibility of their use for mechanical reparation of the heart muscle.

## Background

Human mesenchymal stem cells (hMSCs) are available from bone marrow, umbilical cord blood and adipose tissue. They are multipotent cells, which can differentiate into specialized tissues, including bone, cartilage, fat, tendon, muscle, and stroma [1,2], and allow autologous transplantation. Several studies have shown that hMSCs are capable to differentiate into cardiomyocytes, smooth-muscle cells, and even endothelial cells under certain conditions [3-7]. MSCs transplantation obviates the need for immunosuppression, even when allogenic stem cells are used, since they do not express class II histocompatibility complex and co-stimulatory molecules required for activation of T-cells [8,9].

Most studies on stem cell transplantation aimed at the treatment of myocardial infarction in animal models and human clinical trials have focused on the use of undifferentiated stem cells, so that cardiomyogenic differentiation would be expected to take place *in vivo *within a transplant recipient. Nonetheless, since undifferentiated MSCs tend to spontaneously differentiate into multiple lineages when transplanted *in vivo *[5,10], it is likely that such uncommitted stem cells may undergo unanticipated differentiation within infarcted myocardium. This can in turn reduce the clinical efficacy of the stem cell transplantation therapy for myocardial infarction. Another major consideration would be the safety of using uncommitted cells for transplantation. Adult MSCs may differentiate into fibroblasts rather then myocytes [10]. This may enhance scar formation, further depressing myocardial function and creating a substrate for life-threatening arrhythmias. There also may be other life-threatening consequences of undifferentiated MSCs transplantation. For example, Forrester et al. [11] observed the sympathetic nerve sprouting, resulting in myocardial sympathetic hyperinnervation in swine that could cause ventricular tachyarrhythmias [11,12]. Thus, it was postulated that a certain cardiac differentiation of stem cells prior to transplantation would result in higher engraftment efficacy, as well as in enhanced myocardial regeneration and recovery of heart function [3,6,7,13].

Since 1999, when Makino et al. first reported that bone marrow mesenchymal stem cells treated with 5-azacytidin are able to differentiate into cardiac cells that spontaneously beat *in vitro *[14], plenty of studies in the field of directed cardiomyogenic differentiation of MSCs have been done. Bone marrow-derived mesenchymal stem cells have been reported to transdifferentiate into cardiomyocytes following treatment with several growth factors (TGFβ1, ILGF, PDGF, bFGF) and nonspecific differentiating inducers (5-azacytidine, dynorphin B, insulin, ascorbic and retinoic acids etc.) [13]. However, the types and characteristics of these stem cells remain poorly defined, and the efficiency of transdifferentiation greatly varies between publications.

We report the results of our complex study on directed cardiac differentiation of hMSCs *in vitro*, in which different published protocols of cardiac-specific differentiation of hMSCs and their modifications were examined to find the most promising one, and to reveal the possible mechanisms of hMSCs transdifferentiation. We attempted to cover all principal trends discussed in literature, such as use of growth factors, chemical inductors, biomaterial scaffolds, and co-culture techniques.

## Results

### Untreated hMSCs

**Figure 1:**
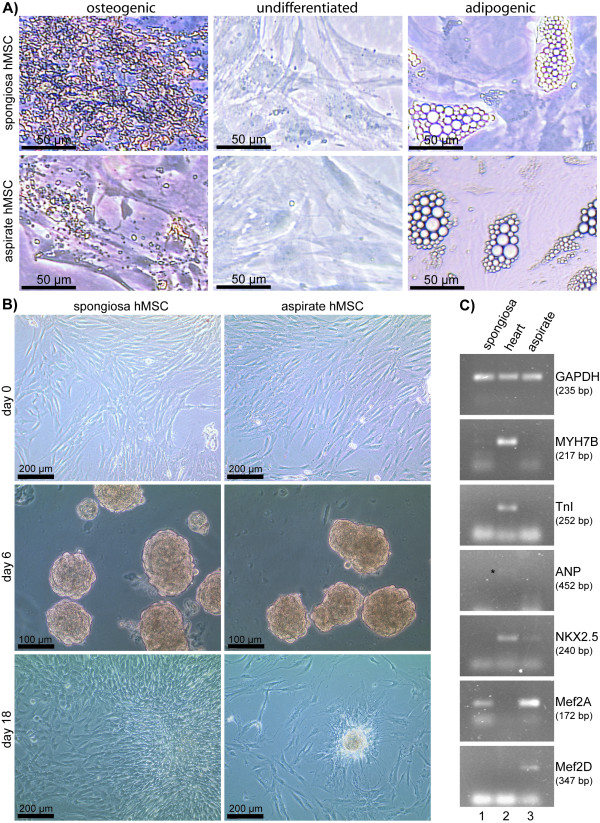
**Spongiosa and aspirate hMSCs cultures**. **(A) **Spongiosa and aspirate hMSCs were cultured in stem cell medium alone (undifferentiated) or in culture medium supplemented with stimuli inducing osteogenic differentiation (osteogenic) and adipogenic differentiation (adipogenic), respectively. In both, spongiosa hMSCs and aspirate hMSCs, mineralization nodules formation confirmed osteogenic differentiation and adipogenic differentiation was confirmed by lipid vacuols. **(B) **Figure shows the photographs of the 3-D hMSCs cultures in differentiation cocktail on two different time points as well as 2-D cultures on starting point of the experiment (day 0). Day 6: spongiosa and aspirate hMSCs cultured on nonadhesive Petri plastic dishes on the day 6 by the method of hanging drops in differentiation cocktail. Day 18: the same cells transferred onto tissue culture plastic; the spongiosa hMSCs culture almost reached 100% confluence while aspirate hMSCs still keep together in the form of bodies and almost do not proliferate. **(C) **PCR from undifferentiated aspirate and spongiosa hMSCs and human adult heart tissue demonstrating the expression of Nkx2.5, MEF2A, and MEF2D genes in undifferentiated aspirate hMSCs and MEF2A and MYH7B in untreated spongiosa hMSCs. The expression of MEF2A and MEF2D genes wasn't revealed and ANP gene expression was negligible in adult human heart tissue (the band marked with black star).

To demonstrate the multipotency of both types of isolated hMSCs used in our experiments, spongiosa hMSCs and aspirate hMSCs, differentiation into adipocytes and osteoblasts was carried out. Depending on the differentiation protocol, induced hMSCs contained lipid vacuoles after adipogenic stimulation, and produced calcium deposits after osteogenic stimulation (Fig. 1A). Control cells (cultured in stem cell medium without differentiation stimuli) retained their stem cell characteristics and did not differentiate spontaneously (Fig. 1A). Untreated spongiosa and aspirate hMSCs from the same donor showed different morphology and cell growth kinetics. The spongiosa hMSCs kept spindle shape over 7 passages and high proliferation rate while aspirate hMSCs used to become spread shape at early passages and demonstrated a considerable slowing down of cells proliferation rate after passages 3-4 already (data not shown). This tendency remained also while using cardiomyogenic differentiation protocols (for example, three-dimensional culture of aspirate and spongiosa hMSCs in differentiation cocktail (DC), Fig. 1B). Surprisingly, when examined untreated cells for expression of cardiac markers, primary hMSCs showed different repertoire of cardiac-specific genes expressed depending on the source of the cells: 40 cycles PCR revealed low levels of Nkx2.5, MEF2A, and MEF2D gene expression in hMSCs isolated from bone marrow aspirate, while primary hMSCs cells from spongiosa expressed MEF2A and in a very low extent MYH7B (Fig. 1C). To our knowledge, it is the first time when the comparison of cardiac-specific gene expression by undifferentiated hMSCs depending on the source of their obtaining (spongiosa or aspirate) was done.

### Aspirate hMSCs cultured in differentiation cocktail or in IMDM, two- and three-dimensional culture

**Figure 2:**
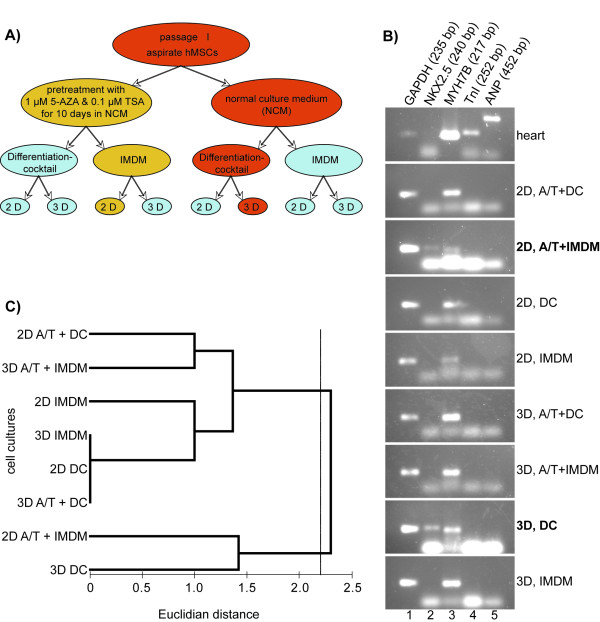
**Test of culture conditions for cardiac differentiation of hMSCs**. **(A) **Scheme of experiment on cardiac differentiation of aspirate hMSCs *in vitro*. **(B) **PCR from human heart tissue and aspirate hMSCs used in 8 differentiation protocols (see A) demonstrating the highest efficacy of two of them that led to the increase in expression of Nkx2.5 gene along with MYH7B gene expression: 3D, DC and 2D, A/T+IMDM. **(C) **Clusterization of cell cultures based on the level of MYH7B, Nkx2.5, MEF2A, and MEF2D gene expression. The cell cultures tested in this experiment formed two statistically different (P = 0.036, ANOSIM) clusters at Euclidian distance of about 2.2. Note that the threedimensional cell culture in DC (3D DC) and the two-dimensional culture in IMDM pretreated with 5-azacytidine and trichostatin A (2D A/T + IMDM) formed a separate cluster. (2D - two-dimensional culture; 3D - three-dimensional culture; A/T - pretreatment with 5-azacytidin and trichostatin A; DC - cells cultured in differentiation cocktail; NCM - normal culture medium; IMDM - NCM based on Iscove's Modified Dulbecco's Medium; Differentiation cocktail - NCM based on DMEM-LG with components of differentiation cocktail).

Aspirate hMSCs were used to test 8 different protocols for cardiac differentiation of hMSCs *in vitro *(Fig. 2A). Immunostaining with antibodies directed against cardiac troponin I, cardiac myosin heavy chain, myoglobin, and smooth muscle actin did not reveal significantly increased expression of cardiac-specific proteins in differentiated cells from all passages, as compared to untreated cells (data not shown).

Nonetheless, cells in three-dimensional culture in medium containing insulin, dexamethasone and ascorbic acid showed elevated levels of Nkx2.5 and cardiac myosin heavy chain (MYH7B) gene expression (Fig. 2B and 2C). The increase in Nkx2.5 expression was also observed in cells pre-treated with 5-azacytidin (AZA) and trichostatin A (TSA) in two-dimensional culture. However, the treatment with AZA is not likely to be applicable "clinically" due to its possible harmful effects. The expression of MYH7B was revealed in all cultures. Nevertheless, the detected negligible levels of Nkx2.5 expression in untreated hMSCs, as well as MEF2A and MEF2D genes (Fig. 1C), present evidence that these markers cannot be considered as an obvious readout of hMCSs cardiac differentiation. Among all examined methods of cardiomyocyte-like cells generation from hMSCs *in vitro*, the three-dimensional cultivation in presence of insulin, dexamethasone, and ascorbic acid (differentiation cocktail) appeared to be the most promising. Probably, the intercellular communication that plays significant role in processes of cell differentiation is much better in three-dimensional culture. Therefore, this protocol was chosen for further studies on spongiosa hMSCs cardiac differentiation *in vitro*. Taking into account that untreated spongiosa hMSCs possess a higher proliferation rate and keep spindle shape in culture much longer then aspirate hMSCs, the spongiosa cells were chosen for further experiments.

### Spongiosa hMSCs cultured in differentiation cocktail, three-dimensional culture

The cardiac-specific gene expression by spongiosa hMSCs cultured in the medium containing chemical inductors of cardiac differentiation (differentiation cocktail, DC) was studied during 3 passages as well as in long term culture on RNA and protein level.

**Figure 3:**
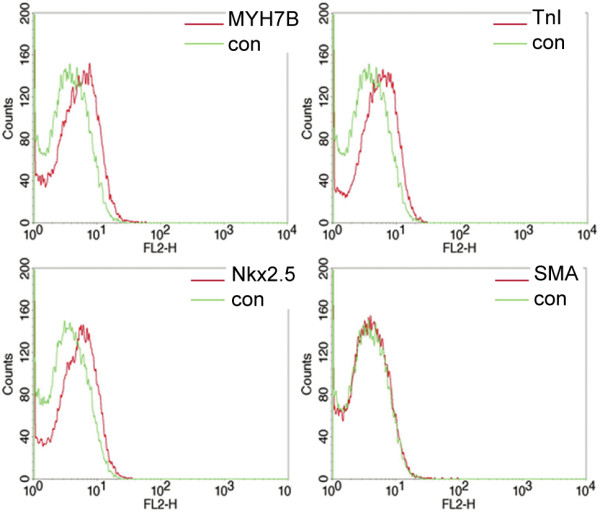
**Expression of cardiac-specific markers by late passage (P3) spongiosa hMSCs cultured in 3-D culture with differentiation cocktail**. FACS analyses of differentiated spongiosa hMSCs stained by antibodies against cardiac myosin heavy chain (MYH7B), cardiac troponin I (TnI), Nkx2.5 and smooth muscle actin (SMA). The slight expression of MYH7B, TnI and Nkx2.5 by all cells and SMA expression by some single cells was revealed.

Flow cytometry revealed a slight increase in expression of cardiac-specific markers (MYH7B, TnI, Nkx2.5) by spongiosa hMSCs cultured in DC at the late passages in comparison to untreated cells (Fig. 3).

**Figure 4:**
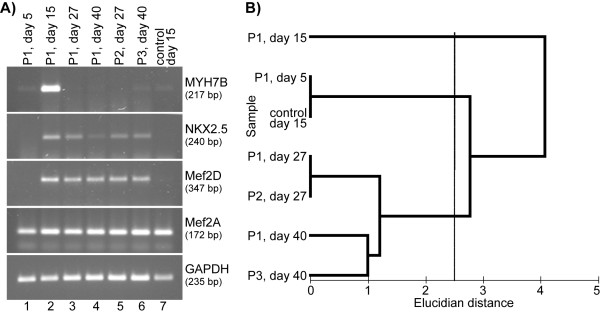
**Cardiac-specific differentiation of spongiosa hMSC cultured 3-D in differentiation cocktail on different time points (passaged cells as well as longterm culture)**. **(A) **PCR from differentiated spongiosa hMSCs demonstrating the temporary pattern of cardiac specific gene expression. The expression of MYH7B and Nkx2.5 was high by the day 15 and then levelled off both in long-term culture and passaged cells. **(B) **Clusterization of cell cultures based on the level of MYH7B, Nkx2.5, MEF2A, and MEF2D gene expression on different time points and passages. The cell cultures tested in this experiment formed three statistically different (P = 0.01, ANOSIM) clusters at Euclidian distance of about 2.5. Note that one of the clusters is formed by first passage MSCs cultured in differentiation cocktail for 15 days (P1, day 15).

PCR analysis revealed increased levels of MYH7B and Nkx2.5 gene expression in cells cultured 3-D by day 15 in DC, followed by a decrease. MYH7B expression levelled off by day 27 (passage 2), but then appeared again by day 40 (passage 3) (Fig. 4A and 4B). The same tendency was clearly seen in long-term 3-D culture of hMSCs in DC (passage 1, day 27 and 40). Currently, we can not explain this observation. The highest level of MEF2D gene expression in 3-D culture was also observed by day 15. Since in 3-D culture untreated spongiosa hMSCs and cells in DC after 5 days were negative for MEF2D, its appearance by day 15 proves the efficacy of the cocktail. High levels of MEF2A gene expression were present in cells throughout all examined passages, as well as in the long-term culture. As it was mentioned above, some cardiac specific genes are already expressed in untreated cells, therefore, the success of a given method can be judged according to the level of their expression in differentiated cells. For instance, the best result of MSCs treatment with DC in 3-D cultures was observed by day 15, as it is seen from the relatively increased levels of MYH7B, MEF2D and Nkx2.5 gene expression, along with unchanged expression of MEF2A gene.

### Spongiosa hMSCs cultured on biomaterials

The extra cellular matrix (ECM) provides a scaffold to which cells can adhere and with which they can interact, and that is required to cluster cells together. These interactions affect a variety of different events, including gene expression, cell proliferation, motility, and differentiation. Different biomaterials could mimic different kinds of ECM. The biomaterial used to cultivate stem cells can potentially influence stem cell proliferation and differentiation in both, positive or negative ways.

**Figure 5:**
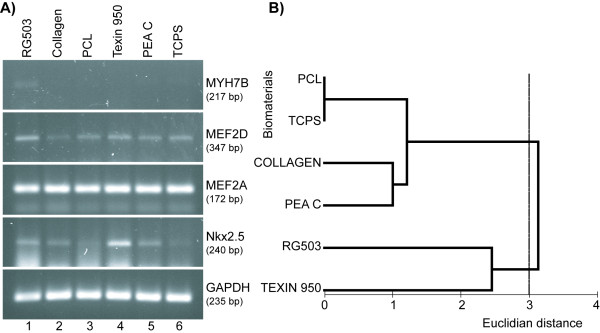
**Expression of cardiac-specific genes by spongiosa hMSCs cultured in DC on biomaterials**. **(A) **PCR from spongiosa hMSCs cultured on biomaterials demonstrating the effectiveness of RG503 and Texin 950 as scaffolds for supporting of cardiac differentiation of hMSCs *in vitro*. **(B) **Clusterization of cell cultures grown on different biomaterials based on the level of MYH7B, Nkx2.5, MEF2A, and MEF2D gene expression. The cell cultures tested in this experiment formed two clusters at Euclidian distance of about 2.8. The level of dissimilarity between these clusters was found to be at the edge of statistical significance (P = 0.06, ANOSIM). Note that the cells cultured on biomaterials RG503 and Texin 950 formed a separate cluster. (TCPS = tissue culture polystyrene).

We have examined the influence of some biomaterials on the efficacy of cardiac differentiation protocol based on the use of differentiation cocktail (2-D culture). Five biodegradable matrices were selected on basis of their compatibility with hMSCs culture judged by cytotoxicity, cell vitality, morphology, apoptosis, and proliferation studies [15]: RG503, Collagen, PCL, Texin 950, PEA C. According to the results of PCR, the most appropriate scaffolds for cardiomyogenic differentiation of hMSCs could be RG503 or Texin 950. Nkx2.5 and MEF2D genes were expressed in cells cultured on all matrices as well as on tissue culture plastic, but the highest levels of expression were observed in cells cultured on RG503 and Texin 950 (Fig. 5A and 5B). The expression of MYH7B was not detected in all cells with the exception of its negligible level in cells cultured on RG503 (Fig. 5A). This phenomenon could be explained by transient pattern of MYH7B expression in spongiosa hMSCs cells cultured in differentiation cocktail as it was previously discussed (Fig. 4A).

### Co-culture of spongiosa hMSCs and Cor.AT cells

Most studies on cardiac transplantation of undifferentiated MSCs relied on the hypothesis that stem cells acquire cardiac phenotype under the influence of local microenvironment, which includes local production of cytokines and growth factors, as well as direct cell-to-cell contact and electrical coupling with native cardiomyocytes. In view of this assumption, we have tried to define the crucial mechanism of such impact. To determine whether direct intercellular communication or molecular substances of cardiac milieu are required for efficient cardiac transdifferentiation of hMSCs, we established direct and indirect co-culture of spongiosa hMSCs and murine atrial-like cardiomyocytes - Cor.AT^® ^cells expressing GFP.

**Figure 6:**
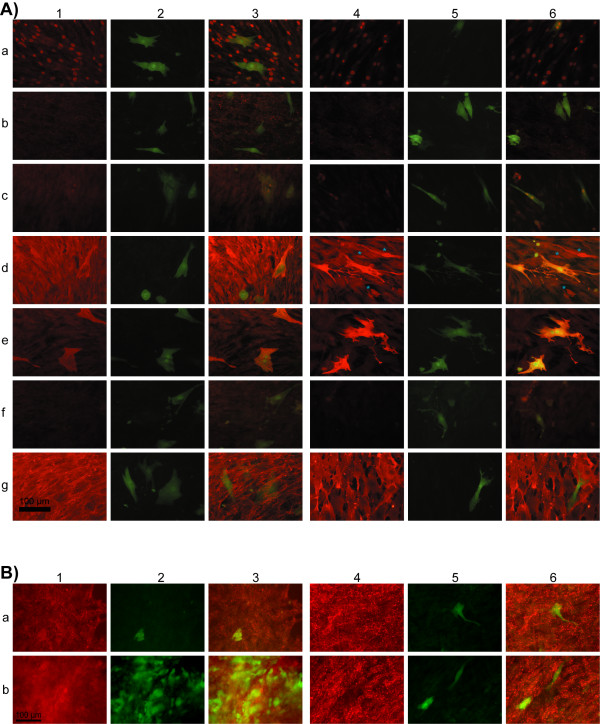
**Expression of myocyte-specific markers by hMSCs co-cultured with Cor.AT cells**. **(A) **14 days co-culture (**1-3**) Co-culture in CorAT medium supplemented with components of differentiation cocktail, (**4-6**) co-culture in Cor.AT medium: 1 and 4 - immunofluorescent staining; 2 and 5 - same field, green coloured cells - murine atrial-like cardiomyocytes (Cor.AT cells) expressing GFP; 3 and 6 - merged. (**a**) GATA4, (**b**) connexin-43, (**c**) Nkx2.5, (**d**) myoglobin (differentiated hMSCs showing organized myoglobin structure are marked with a blue star), (**e**) cardiac troponin I, (**f**) cardiac myosin heavy chain, (**g**) CD44. Scale bar is 100 μm. **(B) **Detection of connexin-43 in 25 days co-culture (**1-3**) Co-culture in CorAT medium supplemented with components of differentiation cocktail, (**4-6**) co-culture in Cor.AT medium: 1 and 4 - immunofluorescent staining; 2 and 5 - same field, green coloured cells - murine atrial-like cardiomyocytes (Cor.AT cells) expressing GFP; 3 and 6 - merged. (**a**) and (**b**) detection of connexin-43 in two independent assays after 25 days. Scale bar is 100 μm.

Immunohistochemical analysis did not reveal significant increase in expression of cardiac-specific markers on days 14 and 25 of co-culturing (Fig. 6). For instance, in contrast to the results of Xu et al. [16] who studied the co-culture of murine bone marrow stem cells and rat neonatal cardiomyocytes, in our experiment hMSCs did not form gap junctions with cardiomyocytes in direct co-culture on day 14 (Fig. 6A: 1b, 3b, 4b, 6b). However, the expression of connexin-43 was detectable both in untreated hMSCs and in hMSCs co-cultured with Co.AT cells on day 25 (Fig. 6B: 1, 3, 4, 6). Nevertheless, the expression of connexin-43 in hMSCs does not prove heart differentiation of stem cells. The detection of connexin-43 in undifferentiated spongiosa hMSCs could be explained by spontaneous upregulation of a wide range of tissue-specific gene expression in hMSCs. For example, mRNA coding for connexin-43 is detectable in undifferentiated spongiosa hMSCs using Chip Array Experiments ("GeneChip^® ^Human Gene 1.0 ST", Affymetrix, data not shown). Apart from connexin-43 no differences in detection of analyzed cardiac-specific markers were observed between day 14 and day 25.

The expression of transcription factor GATA4 was detected in hMSCs and was clearly located in nuclei (Fig. 6A: 1a, 3a, 4a, 6a). The intensity of fluorescence signal given by murine cardiomyocytes nuclei labelled for Nkx2.5 was stronger then by hMSCs nuclei (Fig. 6A: 1c, 3c, 4c, 6c). The expression of myocytes-specific marker myoglobin was high in all hMSCs, but only in some hMSCs co-cultured with Cor.AT cells in Cor.AT medium showed organized structure (Fig. 6A: 4d and 6d; marked with a blue star). Cardiac troponin I with clear structural organization pattern was expressed only by cardiomyocytes and had diffuse distribution in hMSCs (Fig. 6A: 1-6e). Human MSCs are not likely to express the cardiac myosin heavy chain (MYH7B) as the immunostaining analysis shows a diffused pattern of the protein distribution, without any organized structure. Murine cardiomyocytes were negative for MYH7B, even though GFP of Cor.AT^® ^cells are under control of the cardiac myosin heavy chain promoter [17] (Fig. 6A: 1-6f). It is likely to be explained by the lack of cross-reactivity of the antibody used in our study against murine MYH7B (mouse monoclonal antibody clone 2F4). All hMSCs kept high levels of lymphopoiesis differentiation antigen CD44 expression while cardiomyocytes were negative for CD44 (Fig. 6A: 1-6g). It is additional evidence that hMSCs do not really differentiate into heart cells, although the expression of some heart specific markers takes place.

## Discussion

The main goal of the present study was to develop an optimal protocol for directed cardiomyogenic differentiation of human MSCs *in vitro*. The main findings of our work are as follows: i) Three-dimensional culture in the presence of insulin, dexamethasone, and ascorbic acid appeared to be the most promising method of cardiomyocyte-like cells generation from hMSCs *in vitro *relied on the use of simple chemical inducers of cardiac differentiation pathways. ii) Pretreatment with 5-azacytidine and trichostatin A does not lead to any significant increase of the efficacy of differentiation protocols. iii) The biomaterials Resomer^® ^RG 503 and Texin^® ^950 are promising for uses as scaffolds in techniques of cardiac-like cells generation from hMSCs *in vitro*. iv) Even untreated hMSCs demonstrate some level of cardiac-specific gene expression, and therefore, co-culturing of hMSCs with cardiomyocytes does not result in a real transdifferentiation of hMSCs. However, the expression of some heart specific markers by hMSCs in co-culture is achievable. v) The increase in expression of cardiac-specific genes by differentiated hMSCs has a transient character and does not prove the true cardiac differentiation.

We found that hMSCs do not generate functionally active cardiomyocytes *in vitro*, although a part of the cells did demonstrate an increased level of the cardiac specific gene expression when treated with differentiation factors, and chemical substances, or co-cultured with native cardiomyocytes. Probably the generation of functionally active cardiomyocytes requires more time and is not possible in frames of *in vitro *experiments.

Physiologically, adult stem cells within the organism are kept in an inactive state and begin to participate in renewal and differentiation processes only after induction by specific stimuli. The hypermethylation and chromatin compaction by histone deacetylation are proved to be important mechanisms by which some gene expression can be silenced in stem cells [18,19]. To push the spontaneous differentiation of hMSCs, we used the combination of AZA (an inhibitor of DNA methylation) and TSA (an inhibitor of histone deacetylases) prior to start of cardiac differentiation protocols *in vitro*. We observed a spontaneous activation of cardiac muscle genes in hMSCs after demethylation and acetylation, but no significant increase of the efficacy of differentiation protocols could be observed. The cells neither formed any regular cross-striations nor showed spontaneous contractions, which are typical for cardiomyocytes. Our results are in contrast to studies by Makino et al. [14] and Nassiri et al. [20]. This discrepancy could arise due to the fact that treatment with AZA may give random results as it depends on such factors as individual characteristics of the cells' donor, organ the material was isolated from, methods of the cells' isolation, etc. On the other hand, Shiota et al. [21] have shown that MSCs may acquire cardiomyogenic potential after treatment with 5-azacytidine only if they are isolated using specific three-step method based on sphere formation [21]. By day 21 after 5-azacytidin administration, the authors observed single ball-like beating cells. Nonetheless, no beating cardiomyocytes could be obtained under identical induction conditions without selection by sphere formation. When used for subsequent transplantation into infarcted hearts, only few of the MSCs engrafted as cardiomyocytes (less then 0.001% of transplanted cells) [21], providing extra evidence that beneficial effect of MSCs transplantation could not arise from their cardiomyogenic potential.

The increase of transcriptional factors Mef2A, Mef2D, and Nkx2.5 gene expression by hMSCs, upon the induction of cardiac differentiation, does not prove their real transdifferentiation as it was discussed in other studies [6,16]. Although the DNA binding regulatory proteins of myocytes-specific enhancer factor-2 (MEF2) family above all are involved into the process of mesodermal precursor cells to myoblasts differentiation and formation of linear heart tube during embryogenesis [22], the Mef2A mRNA is ubiquitously expressed, with highest levels found in skeletal muscle, heart and brain [23]. Moreover, Mef2A were shown to play a crucial role during nervous system development [24]. The cardiac homeobox transcription factor Nkx2.5 is essential in cardiac development, homeostasis, and survival of cardiac myocytes. Although the expression of Nkx2.5 is mainly restricted to the heart, its non-cardiac functions were also proved [25]. All mentioned above suggests that the expression of MEF2A, MEF2D, and Nkx2.5 cannot be used as an indication for the induction of a cardiac expression program, but these transcriptional factors are still necessary for cardiac differentiation of stem cells. The emphasis should however be placed on the expression of cardiac proteins and functional activity of cells.

It becomes apparent that differentiation of hMSCs depends on stochastic events and is arrested prior to terminal differentiation, probably due to the absence of critical determination events. To check the hypothesis that cell-to cell contact with primary cardiomyocytes or molecular signals exerted by cardiac cells within the transplanted heart may drive the cardiac differentiation of hMSCs, we established a direct and indirect (data not shown) co-culture of hMSCs with murine atrial-like cardiomyocytes (Cor.AT^® ^cells). We failed to achieve a real transdifferentiation of hMSCs, although the expression of some heart specific markers by hMSCs was high in direct co-culture with cardiac cells. Human MSCs in our study did not form the gap junctions with primary heart cells. Extremely rare hMSCs showed an organizational pattern of the tested cardiac intercellular proteins. Despite a robust expression of myosin heavy chain gene, we did not detect organized sarcomeric structures. Similarly, expression of the cardiac TnI was detected by immunofluorescence and PCR but no organized contractile apparatus or cross-striations were apparent.

Our results are in accordance with results obtained by Bedada et al. [26], who succeeded in turning on various cardiac specific markers via different induction regimens but failed in obtaining fully differentiated, functional cells. The authors demonstrated the preferential increase of cardiac gene expression in adult bone marrow derived cells after activation by Wnt11 molecules or treatment with AZA and TSA, including the cardiac TnI, GATA4, Hand-2, and beta-MHC (beta-myosin heavy chain). However, they were unable to identify reproducible expression of other typical cardiomyocyte genes, such as the alpha-MHC and ANP (atrial natriuretic protein) genes [26].

Detection of the cardiac specific gene expression in untreated hMSCs in our study allows us to assume the pre-existing plasticity of hMSCs but, at the same time, it makes doubtful the reliability of this cardiac differentiation criterion. Previous successful studies devoted to cardiac differentiation of hMSCs *in vitro *did not use the expression level of cardiac-specific genes by untreated cells as the negative control [6,16]. Thus, our results let us assert that the majority of previously published data on the directed cardiac differentiation is not reproducible.

The discrepancies between different published data could also arise due to heterogeneity of the MSCs population in bone marrow. The procedures of MSCs isolation and characterization might vary from one lab to another. Since the stroma consists of various mesenchymal cell types, the variability in parameters used for cell sorting and adherence properties of these cells to culture plastic, as well as subsequent culture conditions and other treatments might lead to the isolation and growth of slightly different cell types with different properties in various assays. For example, Bedada et al. [26] have shown that bone marrow stromal cells can differ in the expression of two popular stem cell markers, i.e., CD34 and Sca-1, without having major differences in plasticity and differentiation potentials. It still has to be clarified whether such differences in phenotype can affect the cardiomyogenic potential of different subsets of MSCs [26].

We suggest that "successful" cardiac differentiation of bone marrow stromal cells in some studies could be achieved due to use of the very scarce hMSCs subpopulation able to transform into cardiac cells. If such a cell population does exist, the protocol(s) for its isolation should be unified and reproducible, and the proof of cardiac transdifferentiation should be more obvious than simple detection of the cardiac-specific gene expression. Moreover, in view of the fact that bone marrow stem cells were shown to integrate in myocardium at very low frequency through cell fusion with resident cardiomyocytes out of the region of scar formation in animal model [27] and have no positive effect on left ventricular function [28], we find it extremely unsafe to draw broad generalizations regarding cardiac potential of MSCs. The dose-escalation contribution of transplanted MSCs into formation of pathological encapsulated structures containing calcifications and ossifications within infarcted myocardium clearly demonstrated by Breitbach et al. [29] further challenges their surrounding tissue-restricted fate. All mentioned above calls for prudence in interpretation of casual reports of successful cardiac transdifferentiation of hMSCs.

Our results provide additional evidence that functional benefit observed after intramyocardial transplantation of MSCs reported in animal studies is likely to be caused by definite positive impact on the left ventricular remodelling and angiogenesis, rather than by direct myocardial regeneration. Numerous studies have been devoted to antiapoptotic, anti-inflammatory, and proangiogenic action of MSCs in the site of cardiac remodeling processes after myocardial infarction. MSCs demonstrate local immunomodulating properties, suppressing the activity of a broad range of immune cells, including N-cells, B-cells, natural killer cells and antigen presenting cells. Transplantation of MSCs into the periinfarct area decreases both production and gene expression of the proinflammatory cytokines TNFα, IL-1β and IL-6. The same suppressing impact can be observed in case of the matrix metalloproteinase-1 (MMP-1) and tissue inhibitor of matrix metalloproteinase-1 (TIMP-1) that lead to attenuation of the cardiac inflammation and pathophysiological remodeling, such as replacement of the infarcted area by scar, myocyte hypertrophy, myocyte loss through apoptosis, alterations of endothelial matrix, and endothelial dysfunction [30]. Besides VEGF (promotes significant angiogenesis) and IGF-I (antiapoptotic effect on cardiomyocytes) [31], MSCs secrete plenty of other paracrine factors that prevent apoptosis, promote angiogenesis and assist in matrix reorganization, e.g., SDF-1 (trophic support of cardiac myocytes and angiogenic effect via promoting of endothelial progenitor cells homing to the site of injury) [32], FGF (angiogenic, antifibrotic, antiapoptotic effect), HGF (angiogenic, antiapoptotic, mitogenic, antifibrotic activities), adrenomedullin (angiogenic, antiapoptotic and antifibrotic activities) [33].

## Conclusion

Summarizing our results, we conclude that real cardiac differentiation of the adult hMSCs is not achievable, although the cells demonstrate high plasticity and respond to induction of the cardiac differentiation pathways in the way of stochastic increase of certain cardiac-specific gene expression. Mesenchymal stem cells should be considered as an ideal tool for gene therapy of ischemic heart disease, or as antiapoptotic immunotherapeutic agents in myocardial regeneration after infarction, rather then crude for mechanical substitution of dead cardiomyocytes.

## Methods

### Isolation of human mesenchymal stem cells

Spongiosa hMSCs were isolated mechanically from the femoral heads of patients with total hip joint endoprosthesis (donations received with informed consent) and by adherence to cell culture plastic according to protocols of Haynesworth et al. [34] and Pittenger et al. [2]. Briefly, the bone marrow spongiosa was rinsed several times with stem cell culture medium consisting of Dulbecco's Modified Eagle's Medium low glucose (DMEM-LG, Sigma, Steinheim, Germany), 10% FCS, 2 mM L-Glutamin, 100 U/ml penicillin, and 100 μg/ml streptomycin (all from PAA Laboratories, Linz, Austria). Spongiosa was then removed and the remaining cell suspension was centrifuged for 10 min at 500 × g. Bone marrow aspirate was resuspended in 20 ml of culture medium, vortexed, and centrifuged for 10 min at 500 × g. Thereafter, the cell pellets were resuspended in 10 ml of medium, and the cells were seeded in 10 cm culture dishes. After 24 hours, non adherent (hematopoietic) cells were removed by medium change. Mesenchymal stem cells were expanded in stem cell culture medium at 37°C in a 5% CO_2 _and 20% O_2 _humidified atmosphere. Medium was changed every 3-4 days. At 80-90% confluence (about 10,000 cells/cm^2^), stem cells were trypsinized with stem cell trypsin (CellSystems, St. Katharinen, Germany) and re-plated at a density of 1000/cm^2 ^to expand cell culture (cell proliferation of about factor 10 per passage). Depending on the donor and the passage the cell doubling times varied for spongiosa hMSCs between 21.5 and 74.7 hours and for aspirate hMSCs between 23.0 and 97.0 hours. Normally, a slowdown of the cell doubling time could be observed after 7-11 passages. Cell doubling time (hours per doubling) was calculated by the equation: 1/[Log (final cell number/initial cell number)/log2] × time in hours. Cells were characterized by flow cytometry, and multipotency was tested using standard protocols according to Pittenger et al. [2]. Human MSCs from passage 2 and later passages were used for differentiation studies.

### Adipogenic differentiation

Cells were seeded at a density of 80,000 cells/cm^2 ^and then cultured alternately in adipogenic induction medium (DMEM-HG, 10% FCS (both from PAA Laboratories, Linz, Austria), 0.2 μM indomethacine (Biomol, Hamburg, Germany), 1 μM dexamethasone, 10 μg/ml insulin, 0.5 mM IBMX (all Sigma, Steinheim, Germany)) and adipogenic maintenance medium (DMEM-HG, 10% FCS, 10 μg/ml insulin). During 3 weeks of induction, medium was changed semi-weekly.

### Osteogenic differentiation

To induce osteogenic differentiation, cells were seeded at a density of 30,000 cells/cm^2 ^and cultured in osteogenic induction medium (DMEM-LG (Sigma, Steinheim, Germany), 10% FCS, 100 nM dexamethasone, 10 mM sodium-β-glycerophosphate, 50 μM L-ascorbic-acid-2-phosphate (all Sigma, Steinheim, Germany)) for 21 days. Medium was changed thrice weekly.

### Cardiomyogenic differentiation *in vitro*

The cardiomyogenic potential of hMSCs isolated from the bone marrow aspirate and spongiosa, was studied during four passages. The published protocol of cardiac differentiation of MSCs in medium containing insulin, dexamethasone and ascorbic acid [6], as well as accepted techniques of cardiac differentiation of embryonic stem cells [35], were adapted for two- and three-dimensional culture of hMSCs. Briefly, spongiosa and aspirate cells of passages 2 to 6 were cultured in differentiation cocktail consisting of 60% DMEM-LG, 28% MCDB-201 (both from Sigma, Steinheim, Germany), 10% FCS (PAA Laboratories, Linz, Austria), 1 mg/ml bovine insulin, 0.55 mg/ml human transferrin, 0.5 μg/ml sodium selenite, 50 mg/ml BSA, 0.47 μg/ml linoleic acid, 100 nM ascorbate phosphate, 1 nM dexamethasone (all from Sigma, Steinheim, Germany), 100 U/ml penicillin G, 100 μg/ml streptomycin (PAA Laboratories, Linz, Austria) or in Iscove's Modified Dulbecco's Medium (IMDM; Sigma, Steinheim, Germany) supplemented with 10% FCS, 100 U/ml penicillin G, 100 μg/ml streptomycin (PAA Laboratories, Linz, Austria), 1% non-essential aminoacids (NEAA), 0.1% β-mercaptoethanol. The initial seeding density was 1500 cells/cm^2^. Long-term cultured as well as passaged cells were analyzed.

Hanging drops: The drops of hMSCs suspension (400 cells/20 μl) in appropriate culture medium (depending on the differentiation protocol) were placed on the lower surface of the lid of plastic Petri dishes filled with PBS and cultured at 37°C in a 5% CO_2 _and 20% O_2 _humidified atmosphere. Within 3 days cells aggregated to form round shaped bodies and then they were washed away with culture medium and transferred into suspension. On day 5 of culture the defined number of bodies were captured and transferred into cell culture dishes and plates (10 bodies per well of 48-well plate, or 128 bodies per 3.5 cm culture dish).

Treatment of cell cultures with 5.-azacytidin (AZA) and trichostatin A (TSA): Passage 2 hMSCs were pretreated with 1 μM 5-AZA and 0.1 μM TSA for 10 days to increase the differentiational potential of hMSCs. The used concentrations were determined using a dose-response curve.

### Co-culture with Cor.AT^® ^cells

Direct co-culture was established by seeding passage 2 spongiosa hMSCs and mouse atrial-like cardiomyocytes (Cor.AT^® ^cells, AXIOGENESIS AG: ) together in 16-well chamber slides (Nunc) freshly coated with human Fibronectin (BD) for 3 hours at 37°C (20000 hMSCs and 20000 Cor.AT^® ^cells per depot). Cells were cultured in CorAT^® ^medium (AXIOGENESIS AG: ) or in CorAT^® ^medium supplemented with components of differentiation cocktail: 1 mg/ml bovine insulin, 0.55 mg/ml human transferrin, 0.5 μg/ml sodium selenite, 50 mg/ml BSA, 0.47 μg/ml linoleic acid, 100 nM ascorbate phosphate, 1 nM dexamethasone (all from Sigma, Steinheim, Germany). Spongiosa hMSCs as well as Cor.AT^® ^cells separately in both kinds of culture media were used as controls. Medium was changed twice a week and cells were used for analysis after 14 and 25 days.

### Biomaterial scaffolds

Five different biopolymers were chosen on the basis of the assessment of their biocompatibility with hMSCs (cytotoxicity of biomaterials, cell morphology, vitality, apoptosis, and cell proliferation were used as criteria) according to the results of previous studies [15]: Resomer^® ^RG 503 (poly (D, L-lactid-co-glycolic acid), Collagen, PCL (polycaprolactone), Texin^® ^950 (aromatic polyether-based thermoplastic polyurethane), PEA C (polyesteramide type C) (German Wool Research Institute RWTH, Aachen, Germany). Cells were plated at 2000 cells/cm^2 ^in differentiation cocktail in 48-well plates containing biopolymer scaffolds on the bottom for two weeks.

### Antibodies and immunostaining

Cells were fixed in Accustain^® ^(Sigma, Steinheim, Germany) for 10 min., permeabilized for 15 min. (0.5% NP40 in PBS), non-specific background was blocked by saturation solution (SS: 10% FCS, 0.1% Tween 20 in PBS) for 30 min. Afterwards, cells were incubated with primary antibodies for 2 h (1:100 in SS). After washes, Alexa Fluor-488- or -546-conjugated anti-rabbit, anti-mouse or anti-rat antibody (Molecular Probes, Gottingen, Germany) was added to the cells for 1 h (1:500 in SS). Signals were visualized using a LEITZ DM IRB fluorescent microscope (Leica) equipped with a digital camera (Nikon), and images were analyzed with Discus-software (Sonja Walther, Munchen, Germany). To characterize cells, antibodies against cardiac troponin I (Abcam), cardiac myosin heavy chain (Upstate), connexin-43 (Alpha Diagnostic), Nkx2.5 (Santa-Cruz), GATA4 (Santa Cruz), myoglobin (Dako), and CD44 (Beckton Dickinson) were used.

### FACS analysis

Cells were enzymatically removed from the culture dish using stem cell trypsin (CellSystems, St. Katharinen, Germany) for 10 min at 37°C, washed with ice-cold sterile-filtered PBS (was used also for all other washes), permeabilized for 15 min. (0.5% NP40 in PBS), resuspended in blocking buffer (10% FCS in PBS) and divided into samples containing 100,000 cells. After 30 min cells were probed for 1 h at room temperature with a 1:500 dilution of first antibodies (mouse anti-human cardiac troponin I, Abcam; cardiac myosin heavy chain, Upstate; Nkx2.5, Santa-Cruz; smooth muscle actin, Dako; or an appropriate isotype control) in blocking buffer. They were then washed in PBS and probed in blocking buffer containing a 1:2000 dilution of FITC-conjugated goat antimouse IgG (Caltag Laboratories, Carlsbad, CA) for 30 min in the dark at room temperature. Cells were then washed 4 times with PBS and fixed with 1% formaldehyde in PBS. The samples were analyzed using FACS-Calibur equipment (Becton Dickinson, San Jose, CA, USA). The cell population of interest was determined and dead cells were excluded using forward and side scatter parameters. Acquisition was set for 25,000 events per sample. The data was analyzed using the Cell Quest Software (Becton Dickinson).

### Reverse transcription and polymerase chain reaction

Total RNA was extracted from cultured cells using RNeasy Kit (Qiagen GmbH, Hilden, Germany). Human and murine cardiac RNA was used as positive control. 100 ng to 1 μg RNA (depending on the amount of cell material in given experiment) was converted to cDNA using Superscript III first-strand synthesis kit (Invitrogen, Carlsbad, CA). PCR was performed with Taq polymerase (Amersham, Buckinghamshire, UK) for 35-40 cycles, with each cycle consisting of denaturation at 95°C for 10 s, annealing at primer-dependent annealing temperature (58°C or 60°C) for 2 min, and extension at 71°C for 2 min, with an additional 6 min of autoextension at 72°C after completion of the final cycle. The PCR products (8 of 25 μl) were size-fractionated by 1.5% agarose gel electrophoresis. PCR primer sequences, expected fragment sizes and optimal PCR annealing temperatures used for human cardiac β myosin heavy chain (MYH7B), myocyte enhancer factor-2D and -2A (MEF2D, MEF2A), cardiac troponin I (TnI), atrial natriuretic peptide (ANP), homeobox protein Nkx2.5 and Glyceraldehyde-3-phosphate dehydrogenase (GAPDH) are listed in Table 1.

**Table 1:** RT-PCR primer sequences, product sizes and annealing temperatures

**Gene**	**Primer sequence**	**Product size, bp**	**Annealing temperature, °C**
hMYH7B	5'-AGCAGAAGCGCAACGCAGAGT-3' 5'-TGCTGCACCTTGCGGAACTTG-3'-	217	60
hMEF-2D	5'-TGCCCACTGCCTACAACACAG-3' 5'-GGACACCGGTTCTGACTTGATG-3'	347	58
hMEF-2A	5'-GCAGCAGCACCACCTAGGAC-3' 5'-CTGCTGCTGCTGCTGGAAG-3'	172	58
hTnI	5'-ACTTGTGCCGACAGCTCCAC-3' 5'-CCTTCTTCACCTGCTTGAGGTG-3'	252	58
hANP	5'-CAACGCAGACCTGATGGATTTC-3' 5'-AGATGACACGAATGCAGCAGAG-3'	452	58
hNkx2.5	5'-CAAGGACCCTAGAGCCGAAAAG-3' 5'-CCTGCGTGGACGTGAGTTTC-3'	240	58
hGAPDH	5'-GAAGGTGAAGGTCGGAGTC-3' 5'-GAAGATGGTGATGGGATTTC-3'	235	58

### Statistics

The results of cardiac specific gene expression analysis were ranked from 0 to 4 in accordance with the intensity of fluorescence stain of RCR products in agarose gel, in relation to the housekeeping gene. Average linkage cluster analysis was then applied to the obtained data matrix using PRIMER6 software (PRIMER-E Ltd., 2006) [36]. The similarity between tested cell cultures was estimated with Euclidian distance. ANOSIM (analysis of similarity) routine of PRIMER6 was applied to test if there was a statistically significant difference between revealed clusters of the cell cultures. The difference was considered significant at P < 0.05.

## Competing interests

The authors declare that they have no competing interests.

## Authors' contributions

BD conceived and designed the experiments. SM performed the experiments. BD and SM analyzed the data. BD contributed reagents/materials/analysis tools. BD and SM wrote the paper.
